# Progression of lower and higher-order aberrations: a longitudinal study

**DOI:** 10.1186/1471-2415-15-11

**Published:** 2015-01-24

**Authors:** Balamurali Vasudevan, Brian Fisher, Barry Case, Phu Lam, Jeff Wayman

**Affiliations:** College of Optometry Midwestern University, Glendale, AZ 85308 USA

**Keywords:** Spherical aberration, Coma, Higher-order aberrations, Myopia, Longitudinal study, Defocus

## Abstract

**Background:**

The aim of the present study was to investigate the effect of near-work on lower and higher-order aberrations, and its progression over a 9-month period during the school year.

**Methods:**

Data from 24 young-adult myopic eyes, and 24 non-myopic eyes were used in this investigation. The lower-order aberrations, coma, spherical aberration (SA), and total root mean square (RMS) of higher order aberrations (total HOA) were measured using an open-field iTrace aberrometer, at both the initial baseline evaluation, and then at the follow-up visits over a 9-month period. Pupil size of 4 mm was used for the aberration measurements.

**Results:**

The group mean (SD) of the subjects (mean age: 23.6 +/- 3.4 years) at the initial and follow-up visit was 0.47D (0.47D) and 0.31D (0.41D), in the non-myopes and -3.58D (2.08D) and -3.86D (2.14D) in the myopes, respectively. Significant increases in myopic refraction were observed. The group mean (SD) total HOA at the initial and final visit was 0.12 (0.08) and 0.11 (0.06) microns, in the non-myopes, and 0.15 (0.08) and 0.15 (0.08) microns, in the myopes, respectively. The group mean RMS of the coma at the initial and final visit was 0.06 (0.04) and 0.07 (0.05), in the non-myopes, and 0.08 (0.06) and 0.09 (0.06) microns, in the myopes, respectively. The group mean SA of the subjects at the initial and last visit was 0.04 (0.04) and 0.03 (0.03), in the non-myopes, and 0.04 (0.04) and 0.04 (0.04) microns, in the myopes, respectively.

**Conclusions:**

There was a significant difference in myopic refraction, over the 9-month assessment period. However, no significant difference in total HOA, SA, and coma between the initial and follow-up visits in both the myopes and the non-myopes was observed.

## Background

Myopia approximately affects 25% of US populations and as much as 80% of the young population in Asian countries [1]. It has been suggested in many studies that the development and progression of myopia is primarily due to environment and genetic factors [2]. In addition, it has been suggested that near work is one of the primary environmental factors causing myopia in children and young adults [3]. Previous studies [4–10] have reported that the prevalence of myopia is much higher in medical and law students, presumably due to the significantly higher near work demand. Jorge, Almeida, and Parafita [11] studied the refractive error progression in 118 Portuguese university students. Following 3 years of academic course work, the mean refractive change for the M, J0 and J45 components was -0.29 ± 0.38 D (p < 0.001), 0.02 ± 0.16 D (p = 0.281), and 0.01 ± 0.09 D (p = 0.784). They reported that the prevalence of myopia increased by 5.1%, while the prevalence of hyperopia decreased by 9.4%, during this period. Furthermore, myopic progression in individuals with emmetropia was observed in 22% of the population. In addition, Jorge et al. [7] performed selected accommodative and binocular vision tests in these university students. Significant changes in near heterophoria, fusional vergences, and positive relative accommodation were found. Interestingly, the break value of the base-in fusional vergence test was found to be a significant predictor of myopic shift in young adults.

Jiang, Schatz & Seger [4] reported that optometric students engaged in visual activities that involved significant periods of near work during their first academic year experienced changes in their refractive error. The myopic changes were most prominent during the semesters with more intense near work. They also reported that this progression was more rapid in myopes than in either emmetropes or hyperopes. Another notable finding was a decrease in myopia, or a reversal towards emmetropia, that occurred after summer vacation, when the students performed much less near work. In a seperate investigation, Septon [12] found that 74.3% of optometry students in their sample were either more myopic or became myopic in the second year of the optometry program. In contrast, Bullimore, Conway, and Nakash [10] reported that 55.6% of the students in the Aston University undergraduate program were myopic or became myopic. This difference could be accounted by the greater near work activities performed by the optometry students versus the undergraduate students. Similar effects have been noticed in medical and law students. While these studies reported on the myopic shift, there has been very little information available regarding the progression of higher-order aberrations and their interactions with retinal defocus in young adults.

In contrast, some studies [e.g., Low, Dirani, Gazzard & Chan [13]] have reported on the lack of effect of near-work on myopic progression. Furthermore, recent investigations have also suggested that children who spend more time outdoors are less likely to become myopic, irrespective of how much near work they do, or whether their parents are myopic [14, 15]. Lastly, Cheng, Himebaugh, Kollbaum, Thibos and Bradley [16] investigated the reproducibility of the optical aberrations with measurements performed at few seconds, 1 week, 1 month, and 1 year apart. There were minor variations in the optical aberrations over all the time period, and it was not clinically significant. Recently, Miranda, O’Donnell, and Radhakrishnan [17] also investigated the repeatability of monochromatic ocular aberrations taken 1 minute, 1 hour and 1 week apart. They too reported relatively little differences.

A systematic evaluation of the changes in refractive state as well as ocular aberrations of the eye as a function of near work has not been documented and performed as a longitudinal study. Thus, the primary aim of the study was to investigate the progression of the refractive error, and ocular aberrations, over a period of nine months.

## Methods

Data from 24 young adult myopic eyes and 24 non-myopic eyes, recruited from the Arizona College of Optometry were used in this investigation. Mean age was 24.3 +/- 3.2 years. Myopic subjects were defined as having a spherical equivalent refraction of-0.5D or higher, and non-myopic subjects (mostly emmetropes and low hyperopes) were defined as having a spherical equivalent refraction of-0.37D or less at the initial screening visit. The upper limit for non-myopes was 1D. In addition, subjects with astigmatism greater than 0.5D were excluded. In subjects with astigmatism between 0.25D and 0.5D, the spherical equivalent was calculated. Any subject with a history of ocular/systemic pathology, refractive surgery, or a binocular vision anomaly was excluded. In addition, subjects with anisometropia of 1D or more were excluded.

Data collection was performed from the right eye only at four different visits during the academic year (final week of August though the 3rd week of May). A questionnaire [18] was used at every visit to determine the magnitude of near work hours spent by each subject within a week, the hours spent performing near work per day was then calculated. Questions included the number of hours spent studying academic material, reading for pleasure, watching TV, and playing video games/computer. Near work hours for each subject varied between the initial and the subsequent visits. Initial visit including baseline screening was performed 1 week prior to the start of the academic program.

During the initial visit, each subject was screened extensively (accommodative and binocular vision tests were performed) to ensure that they would be eligible for the study. Upon completion of the eligibility screening, subjects were scheduled for the initial visit in the same week. During the initial visit, objective refraction and optical aberrations were determined from both the eyes using iTrace aberrometer, and furthermore, unaided retinoscopy was performed to determine the initial refractive state. Non-cycloplegic subjective refraction was also performed at each visit. It was used to determine the refractive state at each visit, and classify subjects as either myopes or non-myopes. Furthermore, post-cycloplegic subjective refraction was performed to only determine the true refractive state (to identify any latent hyperopia and rule out pseudomyopia) of the eye at the screening and the final visit, and not used for any of the analysis. Spectacles or contact lenses habitual correction by the subjects was not used to classify the subjects as myopes or non-myopes. Measurements obtained in the first visit were also repeated at the 9th month follow-up (final) visit.

Three measurements of monochromatic optical aberrations were obtained and averaged using an iTrace from the right eye with a natural pupil size (non-cyclopleged). Subject’s primary line of sight was aligned with the iTrace measurement axis and the subjects were instructed to fixate a 20/200 letter target under binocular conditions placed 20 ft away (to minimize any accommodative changes). Subjects were able to identify the fixation target and maintain fixation when the measurements were gathered. Prior to taking the measurements, subjects were advised not to do any near work activities. Subject’s refractive error was not corrected when the iTrace measurements were obtained, but were able to maintain fixation on the fixation target. Room illumination was kept to normal standard lightning levels, and was modified using a dimmer switch. Light level was modified for each subject until the pupil size became 4 mm. All the measurements were obtained under photopic conditions. Subjects were instructed to remain stationary and fixate the target with the chin in the chinrest and forehead rested while maintaining normal blinking. Immediately before each measurement, subjects were instructed to blink, and then held their eyes open. Aberrations were taken approximately 1 second after the final blink. Higher-order optical aberrations measured using iTrace included total HOA, RMS of coma and SA at 4 mm pupil size in all the subjects. Measurements were obtained at 4 mm as it represents normal pupil size under typical indoor lighting conditions.

Each of the follow-up visits took approximately 60-70mins to be completed and were performed at the last week of the academic program to ensure that maximum near work effect could be generated. Data from the initial and the final visit is only reported.

Written informed consent was obtained from all the subjects only prior to the participation in the study. The study protocol was approved by the Midwestern University IRB committee. The research adhered to the tenets of the Declaration of Helsinki.

Only non-cycloplegic refractions were used for analysis. Although cycloplegic refraction was also obtained at the baseline and the final visit, it was used only as a confirmatory test to ensure that the refractive error change noticed was a true effect. Normality of all data distributions was confirmed using the Kolmogorov-Smirnov test. Data analysis were performed using paired *t*-test, ANOVA and multiple correlation analysis. All the analysis was performed using SPSS (version 20). Spherical equivalent was used for all the analysis. Difference in spherical equivalent was calculated between the final and initial visit was also calculated and used in the analysis.

## Results

The total number of near-work hours was calculated, and there was a significant difference between the initial and final visit within the myopic (p < 0.05) and the non-myopic (p < 0.05) group. In addition, there was a statistically significant effect between the myopic and non-myopic group at the final visit (p < 0.05) only. At the initial visit, the mean magnitude of near work was 2.8 and 2.3 hours per day for myopes and non-myopes, respectively, while at the final visit, it was 8.5 and 10.3 hours per day for the myopes and non-myopes, respectively (Table 1). Subjects reported that they took a break approximately every hour after near work.

**Table 1:** **Summary of the mean hours spent by the student on various activities during the day**

Activities	Myopes (I)	Non-myopes (I)	Myopes (F)	Non-myopes (F)
**Studying**	0.5	0.5	8	6
**Reading for pleasure**	0.5	0.5	0.5	0.5
**Watching TV**	1	1	0.8	1
**Playing video games**	0.8	0.5	1	1
**Total**	2.8	2.5	10.3	8.5

I = initial visit and F = final visit.

The group mean (SD) spherical equivalent of the subjects at the initial and the follow-up visit for the non-myopes and myopes is given in Table 2. The group mean difference in spherical equivalent of refraction between the initial and follow-up visit for non-myopes and myopes is given in Table 2. The difference in refraction between the baseline and final visit was calculated for myopes and non-myopes, and there was no significant correlation between them (r = 0.21, p > 0.05). Mean astigmatism for myopes was-0.67D at the initial visit, and decreased to-0.55D at the follow up visit. Mean astigmatism for non-myopes was-0.42D at the initial visit, and decreased to-0.37D at the follow up visit.

**Table 2:** **Summary of optical aberrations (in microns) and spherical equivalent (in D) in myopic and non-myopic subjects at the initial and final visit**

	Non-myopes (Initial)	Non-myopes (Final)	Non-myopes (difference)	Myopes (Initial)	Myopes (Final)	Myopes (difference)
	Mean+/-SD	Mean+/-SD	Mean+/-SD	Mean+/-SD	Mean+/-SD	Mean+/-SD
**HOA**	0.12 (0.08)	0.11 (0.06)	-0.01 (0.01)	0.15 (0.08)	0.15 (0.08)	-0.01 (0)
**Coma**	0.06 (0.04)	0.07 (0.05)	0.01 (0.01)	0.08 (0.06)	0.09 (0.06)	0.01 (0)
**SA**	0.04 (0.04)	0.03 (0.03)	-0.01 (0.01)	0.04 (0.04)	0.04 (0.04)	-0.01 (0.01)
**SE**	0.47 (0.47)	0.31 (0.41)	-0.16 (0.06)	-3.58 (2.08)	-3.86 (2.14)	-0.28 (0.07)

SA: spherical aberration, SE: spherical equivalent, SD: standard deviation. Pupil size of 4 mm was used for the measurement of optical aberrations. All the measurements were obtained under non-cycloplegic conditions. Numbers are rounded to the second place of decimal.

Paired *t*-test and Pearson correlation analysis performed between the initial and follow-up visits on spherical equivalent revealed significant increase in myopic refraction in both non-myopes (p = 0.04, t = 2.04; r = 0.69, p < 0.01), and myopes (p < 0.01, t = 3.95; r = 0.99, p = <0.01). ANOVA revealed a significant increase in refractive error within both non-myopes and myopes at final visit (all p < 0.05). However, none of the non-myopes became myopic in the 9-month follow-up period. In addition, correlation analysis performed between the baseline refraction and increase in refractive error change was 0.13 (p = 0.53) for non-myopes and-0.04 (p = 0.82) for myopes, they demonstrated no significant difference. Paired *t*-test between astigmatism at the baseline and the final visit revealed no significant difference in non-myopes (t = 0.61, p = 0.55) and myopes (t = 0.62, p = 0.54).

Power analysis using G-power (version 3.1.9) was performed. The difference between two independent means was used to check if the sample size was sufficient for comparing the refractive error and optical aberrations between myopes and non-myopes. Based on the analysis, a power value of 0.99 was noted at both the initial visit and the final visit. In addition, difference between two dependent means was used to check if the sample size was sufficient for comparing the refractive error between first and final visit in myopes and in non-myopes. In the non-myopes, a power value of 0.86, and in the myopes, a power value of 0.99 was noted.

**Figure 1:**
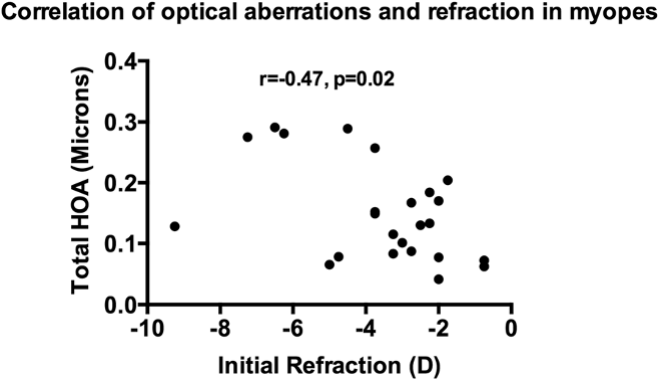
**Plot of correlation of initial refraction and total HOA in the myopes at the baseline visit.** A significant effect was noted.

**Figure 2:**
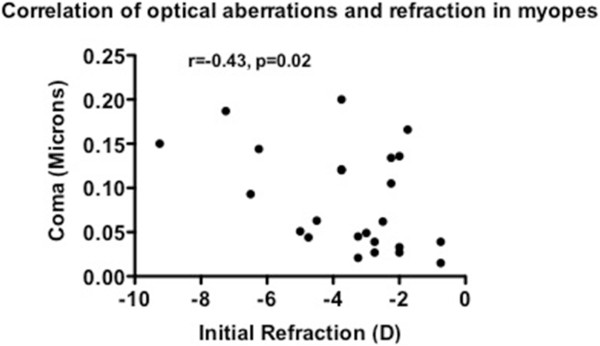
**Plot of correlation of initial refraction and coma in the myopes at the baseline visit.** A significant effect was noted.

**Figure 3:**
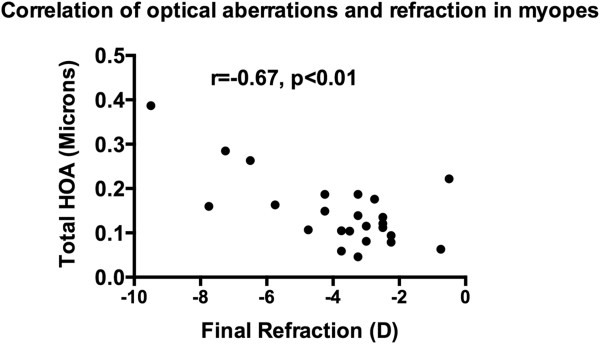
**Plot of correlation of final refraction and total HOA in the myopes at the final visit.** A significant effect was noted.

**Figure 4:**
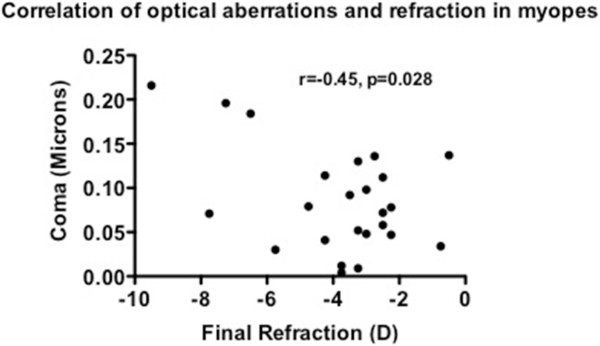
**Plot of correlation of final refraction and coma in the myopes at the final visit.** A significant effect was noted.

The group mean (SD) total HOA of the subjects at the initial and the follow-up visits was 0.12 (0.08) and 0.11 (0.06) microns, in non-myopes, and 0.15 (0.08) and 0.15 (0.08) microns, in myopes, respectively (see Table 2). The group mean (SD) RMS of coma of the subjects at the initial and follow-up visits was 0.06 (0.04) and 0.07 (0.05) microns, in non-myopes, and 0.08 (0.06) and 0.09 (0.06) microns, in myopes, respectively (see Table 2). The group mean (SD) SA of the subjects at the initial and follow-up visits was 0.04 (0.04) and 0.03 (0.03) microns, in non-myopes, and 0.04 (0.04) and 0.04 (0.04) microns, in myopes, respectively (see Table 2). The group mean (SD) difference in total HOA, RMS of coma and SA between initial and follow-up visits in non-myopes was-0.01 (0.01), 0.01 (0.01) and-0.01 (0.01) microns, and in myopes was-0.01 (0), 0.01 (0) and-0.01 (0.01) microns, respectively.Multiple correlation analysis between optical aberrations and refraction was performed in both the non-myopes and myopes. It revealed that there was a high correlation observed in the following relevant conditions in the myopes only: initial refraction and initial total HOA (r = -0.47, p = 0.02; Figure 1), initial refraction and initial RMS of coma (r = -0.43, p = 0.02; Figure 2), final refraction and final total HOA (r = -0.67, p < 0.001; Figure 3), and, final refraction and final RMS of coma (r = -0.45, p = 0.028; Figure 4). This effect was not seen in non-myopes, and this may be due to the relatively restrictive range in refraction. There was no correlation between any of the other optical aberrations and refraction both at the initial and final visit (all p >0.05). In addition, there was no significant correlation between the difference in refraction and the difference in spherical aberration in the non-myopes (r = -0.30, p > 0.05) and the myopes (r = -0.12, p < 0.05).

ANOVA was performed to analyze the presence of any significant change in optical aberrations of the eye between the initial and final visit in both myopes and non-myopes. There was no significant difference in total HOA, RMS of coma, and SA between the initial and final visit in myopes. Similarly, no significant difference was observed for any of the optical aberrations in the non-myopes between the initial and final visit.

## Discussion

There were several interesting and important findings in the present study. First, there was a significant increase in myopic refraction in both the myopes and the non-myopes. Second, there was no significant difference in the optical aberrations between the myopes and the non-myopes. Third, a significant correlation was observed in the myopes for refraction at the initial visit and the following pair of optical aberrations: total HOA and RMS of coma at initial visit. Similarly, significant correlation was observed in myopes for refraction at the final visit and the following pair of optical aberrations: total HOA and RMS of coma.

Defocus and optical aberrations are important components of optical correction of the eye. Both these components vary between individuals and could vary over time. Prior investigations [2] have demonstrated a strong relation between myopia and near-work with excessive academic activity. In addition, several studies have reported on the longitudinal progression of refractive error. A previous study by Jiang, Schatz & Seger [4] reported on the magnitude of myopia progression as a function of dark refraction over the course of 1 year of intense near work in an academic setting. The average myopic refraction progressed by-0.37D within this time frame. Interestingly, the dark focus of refraction was reduced following the summer quarter with a lack of extensive near work. This study reported on the relation between near work and myopia progression in optometry students. The current investigation, performed on a similar cohort of students with similar near task demand, reported a slightly lower increase in myopia progression. Myopes demonstrated a-0.28D increase, while non-myopes demonstrated a-0.15D increase in myopic refraction. Both myopes and non-myopes exhibited a significant increase in spherical equivalent of refraction as a function of increased near work. While this study reported on the myopia progression from the beginning of the fall quarter to the end of the spring quarter, it did not assess the changes in spherical equivalent of refraction in the summer quarter. Near work performed for either shorter or longer durations will have a significant effect on myopia progression. Hung and Ciuffreda [19] reported on the possibility of an incremental retinal defocus theory arising from repeated periods of near work as a contributory factor for an incremental growth of myopia. Experimental evidence has demonstrated the presence of additivity of short durations of near work becoming cumulative over a period of time to produce an increased myopic refraction [20]. A longitudinal study investigating the relation between short durations of near work and permanent myopia is currently ongoing in Beijing, China that is assessing this important relation [21].

Previous studies [22–24] have reported on the temporal variation of the optical aberrations of the eye. Cheng, Himebaugh, Kollbaum, Thibos & Bradley [16] investigated the aberration measurements five times on each day, at the same time of day on five consecutive days, and repeated the same on 5 days at monthly intervals. They reported that there were minor variations in the optical aberrations over all the time period tested, and it was not clinically significant. More recently, Miranda, O’Donnell, Radhakrishnan [17] studied the variation in corneal and ocular higher-order optical aberrations for a shorter duration of 1 minute, 1 day and 1 week obtained from a natural pupil size of 4 mm in a total of 23 subjects. They reported that there was no significant difference between the optical aberrations of the eye using either the Shack-Hartmann aberrometer or Scheimpflug photography. In addition, there was no significant relationship between the age and the variance of corneal and ocular aberrations. Thus, these two investigations have demonstrated that there is no significant increase in optical aberrations over a period of time. Nevertheless, the changes that were reported in these studies could be due to the tear film changes [25]. The results from the present investigation also demonstrated that there were so significant changes after 9 months of follow up.

The relation between the higher and the lower-order optical aberrations of the eye has often been investigated and there has been a mixed response. Studies have reported that there has been an increase in higher-order aberrations with increase in myopia [26, 27], while there are other published investigations that were not able to replicate this effect [28, 29] Similar trend has also been observed with coma. It has been reported that coma is higher with more myopic eye [27], however, a lack of effect was reported by Paquin in Singapore Children [28].

In the eye, there is a constant interaction of lower and higher order aberrations for different tasks under different conditions. Any abnormality or deprivation during emmetropization process could possibly produce a change in the shape of the eye that could change the refractive error. It is well known that retinal defocus is myopigenic in nature [2]. Hyperopic defocus has been reported to be myopigenic in nature in animal models [30], however, newer investigations have proposed the possibility of myopic defocus becoming myopigenic in humans [19]. While blur is the primary drive for accommodation [31], monochromatic aberrations have also been identified as possible cues for accommodation. It is not clear if monochromatic aberrations of the eye have a significant and direct influence on the myopia progression in an individual. Interestingly, few studies [32, 27] have reported that myopic eye typically has larger SA than the emmetropic eye. However, in contrast, other studies [33, 28] have reported that there is no relation between them. Interestingly, Kwan, Yip & Yap [34] reported that larger myopic eyes have smaller magnitudes of SA. In the present study, there is a high correlation observed in the myopes between initial refraction and initial total HOA, initial refraction and initial RMS of coma, final refraction and final total HOA, and final refraction and final RMS of coma. While the optical aberrations investigated in this study did not change significantly over the period investigated, they have been consistently correlated well with the refractive error change and may possibly play a role in myopia development. A large-scale investigation would be needed to better understand this.

How could optical aberrations influence myopia development? Many studies have investigated the effect of higher order aberrations on myopia development (See Charman [35] for a review). It is not clear if these optical aberrations occur before or after the onset of myopia. Positive SA decreases in magnitude and becomes more negative at higher levels of accommodative stimulus. So, individuals with larger magnitudes of myopia could possibly have lesser positive SA or greater negative SA. Kwan, Yip & Yap [34] reported that the more myopic eyes with longer axial lengths demonstrated significantly smaller total higher-order, third- order and SA than less myopic eyes. Larger magnitudes of SA could increase the depth-of-focus of the eye, which in turn could cause a decrease in the accommodative response. When these two mechanisms are taken together, it can be assumed that in a myopic individual with large magnitudes of aberrations, there could be significantly blurred point spread function and a degraded retinal image which eventually could lead to the further development of myopia. Most of the experiments performed so far have not been conclusive on this [35]. It is expected that a longitudinal study on natural history of myopia progression and changes in optical aberrations could possibly address this complicated issue. The current study is one of the first steps in that direction. Future longitudinal studies of at least 3 years should be designed to better understand this relationship. In addition, the role played by peripheral aberration should also be studied.

## Conclusions

There was a significant difference in myopic refraction, however, no significant difference in total HOA, SA, and coma between the initial and follow-up visits in both myopes and non-myopes were observed.

## Abbreviations

SA: Spherical aberration
RMS: Root mean square
HOA: Higher order aberrations.
